# Acoustic Anomalies and Fast Relaxation Dynamics of Amorphous Progesterone as Revealed by Brillouin Light Scattering

**DOI:** 10.3390/ma10121426

**Published:** 2017-12-14

**Authors:** Tae Hyun Kim, Hyojong Yoo, Jae-Hyeon Ko

**Affiliations:** 1 Agency for Defense Development, P.O. Box 35, Yuseong, Daejeon 34186, Korea; taehyunkim@add.re.kr; 2 Department of Chemistry, Hallym University, Chuncheon, Gangwon-do 24252, Korea; hyojong@hallym.ac.kr; 3 Department of Physics, Hallym University, Chuncheon, Gangwon-do 24252, Korea

**Keywords:** progesterone, pharmaceutical, glass, Brillouin scattering, acoustic

## Abstract

The amorphous state of pharmaceuticals has attracted much attention due to its high bioavailability and other advantages. The stability of the amorphous state in relation with the local molecular mobility is important from both fundamental and practical points of view. The acoustic properties of amorphous progesterone, one of the representative steroid hormones, were investigated by using a Brillouin inelastic light scattering technique. The Brillouin spectrum of the longitudinal acoustic mode exhibited distinct changes at the glass transition and the cold-crystallization temperatures. The acoustic dispersions of the longitudinal sound velocity and the acoustic absorption coefficient were attributed to the fast and possibly the secondary relaxation processes in the glassy and supercooled liquid states, while the structural relaxation process was considered as the dominant origin for the significant acoustic damping observed even in the liquid phase. The persisting acoustic dispersion in the liquid state was attributed to the single-molecule nature of the progesterone which does not exhibit hydrogen bonds in the condensed states.

## 1. Introduction

Amorphous pharmaceuticals have attracted great attention because of their potential advantages, such as better bioavailability due to higher solubility, easy production and packaging, etc. [1,2]. They do not have any drawbacks caused by polymorphism, i.e., varied bioavailabilities due to two more crystallographically distinct structures. Each polymorph usually has its unique dissolving rate and different physicochemical properties. However, glassy pharmaceuticals in the nonequilibrium state have higher free energy than crystalline ones. It indicates that aging, i.e., time dependence of physical properties, or recrystallization may limit the shelf life of pharmaceuticals [3,4]. Local molecular mobility remnant in the glassy and supercooled-liquid states might trigger or facilitate the recrystallization process. In this respect, it is important to investigate molecular mobility in their amorphous and ultraviscous (supercooled liquid) states in detail as a function of temperature [5].

Various experimental methods have been used to measure the molecular mobility and relaxation processes at various temporal and spatial measurement scales. For example, dielectric spectroscopy is one of the powerful tools to investigate different relaxation processes of amorphous pharmaceuticals during their transformations in molecular conformation [6,7]. Other experimental techniques have also been adopted to probe dynamic characteristics of amorphous pharmaceuticals [2]. Especially, it is important to measure both local and macroscopic properties in a variety of frequency ranges as wide as possible because the vitrification process is usually accompanied by rich dynamic features such as structural relaxation process, secondary Johari-Goldstein process and other fast relaxation processes. Moreover, better understanding of the amorphous pharmaceuticals may extend our insights into the universality of various glass-forming materials.

Progesterone (C_21_H_30_O_2_, molecular weight: 314.46), which belongs to a group of progestogens, is one of the steroid hormones naturally secreted by the ovary during the menstrual cycle. It has been used for birth control, menopausal therapy, prescription for pregnancy loss or infertility problem, etc. Two orthorhombic polymorphs, Form 1 and Form 2, have been characterized at room temperature [8,9]. These polymorphs show the same space group (P2_1_2_1_2_1_) but different lattice constants [10]. A variety of crystal syntheses and structural characterizations for the polymorphs of progesterone have been performed [10,11,12,13,14,15]. The solvent-mediated transformation between the two crystal structures was investigated by in situ Raman spectroscopy [12]. Form 1 was found to be thermodynamically stable compared to Form 2 [10]. In spite of rather extensive works on crystalline progesterone, little is known about the amorphous state of progesterone. The thermal and dielectric relaxation properties of amorphous progesterone [6] and the equimolar benzoic acid-progesterone mixture [7] were reported. Johari et al. showed that the amorphous progesterone exhibits prominent *α*-relaxation process and secondary Johari-Goldstein relaxation process in the supercooled liquid and glassy state, respectively. The relaxation time of the main *α*-relaxation process showed a diverging behavior satisfying the so-called Vogel-Fulcher-Tammann equation [6]. However, there has been no detailed result about the secondary or fast relaxation processes of amorphous progesterone since the frequency range of these dielectric studies is somewhat limited. The secondary or fast relaxation processes may trigger the crystallization process in the glassy state, thus the detailed investigation by using high-frequency probes is required.

Brillouin spectroscopy is a well-established inelastic light scattering technique that probes the long-wavelength acoustic waves in the GHz frequency range in condensed matter [16]. By observing the energy (or frequency) of the acoustic waves, the sound velocities and absorption coefficients can be easily obtained with high accuracy. Moreover, these acoustic waves can couple to fast relaxation dynamics, the characteristic frequency of which is in the GHz~THz range. Previous Brillouin scattering studies on aspirin [17,18,19], ibuprofen [20,21,22], acetaminophen [23], and other pharmaceuticals [24,25,26] revealed clear anomalies near the glass transition temperatures (*T_g_*) and fast relaxation processes that coupled to the longitudinal acoustic waves. The present study is aimed at investigating and comparing acoustic properties of progesterone in its glassy, ultraviscous, crystalline, and liquid states. Especially, correlation between the acoustic anomalies and various relaxation dynamics in the glassy and ultraviscous states are examined and discussed.

## 2. Results

### 2.1. Brillouin Scattering Spectrum

**Figure 1:**
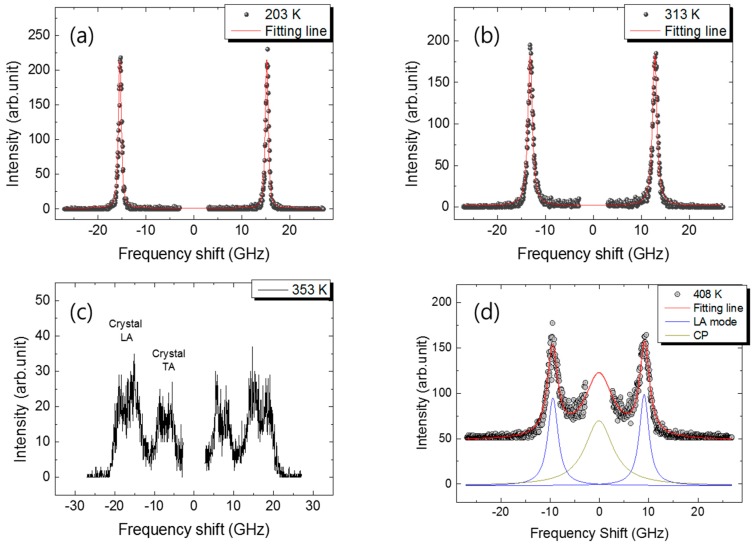
Brillouin scattering spectra corresponding to (**a**) the quenched glassy state; (**b**) the ultraviscous (or supercooled liquid) state; (**c**) the crystalline state and (**d**) the liquid state. Each state was attained sequentially upon heating. The solid lines denote the best-fitted results as described in the text.

The progesterone crystals were melted at 408 K, which is above the melting point (*T_m_* ~ 403 K), and then quenched to 77 K to generate amorphous or glassy state. The glassy progesterone was heated and the Brillouin spectrum was measured at a step of 5 K. Figure 1a–d show Brillouin scattering spectra measured at selected temperatures. These spectra correspond to the states of progesterone starting from the quenched glassy state (203 K) to the ultraviscous state (313 K) and then to the crystalline state (353 K) and finally to the liquid state (408 K). Each state was attained sequentially upon heating. The spectra in Figure 1a,b consist of Brillouin doublets which correspond to the longitudinal acoustic (LA) wave in progesterone. The transverse acoustic (TA) wave cannot be probed at the present backscattering geometry according to the Brillouin selection rule for isotropic materials [27]. The same rule applies to the liquid state, as shown in Figure 1d, where only one Brillouin doublet is observed. The LA mode frequency in the liquid phase is closer to the laser frequency, and its half-width is much larger than that in the glassy state. In addition, a central peak (CP) is observed from the liquid sample, which may be attributed to the Mountain peak reflecting some relaxation effects in the liquid phase [16].

On the other hand, several Brillouin peaks are observed from the crystallized progesterone as shown in Figure 1c. The high-frequency peaks above 10 GHz correspond to the LA mode, while the low-frequency peaks to the TA mode. There are basically three acoustic mode branches near the center of the first Brillouin zone in crystals: one LA and two TA modes. Because of the birefringence effect inherent in the orthorhombic symmetry, each mode is split into two or three peaks depending on the experimental conditions. Furthermore, the crystalline progesterone consists of randomly-oriented crystal grains. Due to the existence of these grains and grain boundaries, the excitation light undergoes multiple refractions and reflections inducing heavy damping of the acoustic waves, and the light directions within each crystalline grain are randomized as well. These effects usually induce distribution of the scattering angle and thus of the Brillouin frequency shift with a high-frequency cut-off corresponding to the frequency shift at the backscattering geometry [28]. The largest Brillouin shift is due to the largest scattering wavevector at the scattering angle of 180°. These superposed LA and TA modes plotted in Figure 1c are due to the multiple scattering events as well as the birefringence effect. Finally, as the crystallization is complete, the crystallized progesterone becomes opaque and further measurement was not possible.

**Figure 2:**
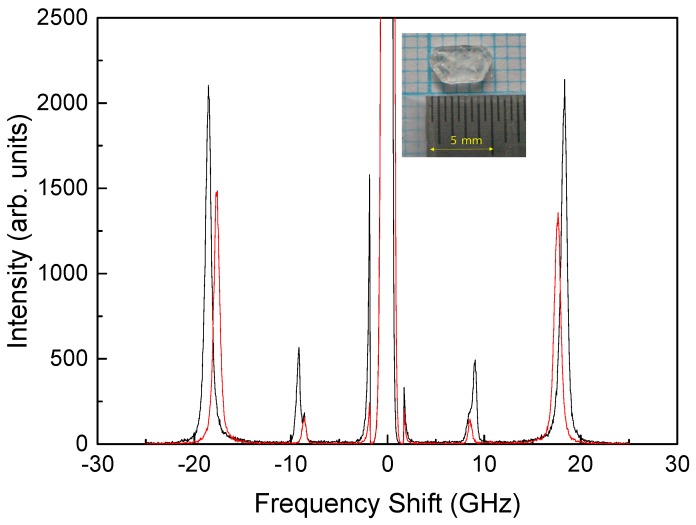
Two Brillouin spectra of the grown progesterone single crystal obtained at two different polarization directions of the laser light incident on the crystal surface. (Inset: a photograph of the grown progesterone crystal).

In order to corroborate the above suggestion, the Brillouin spectrum of the grown progesterone was measured independently. Figure 2 shows the Brillouin spectra of the grown single crystal. The inset shows a photograph of the grown progesterone crystal. Its crystal morphology is similar to the reported one [11]. Two Brillouin spectra were obtained at two different polarization directions of the light incident on the crystal surface at the backscattering geometry. The crystal axes were not identified in this measurement because the main purpose of this investigation was to check the approximate frequency ranges of the LA and the TA modes. Figure 2 shows the LA and TA mode frequencies at the backscattering geometry, where the frequency shift becomes largest, located at ~19 and ~9 GHz, respectively. Because of the multiple refractions at the grain boundaries, the scattering angle is distributed between 0–180°. This is the reason why the distributed LA and TA mode frequencies are observed as shown in Figure 1c. In particular, the approximate cut-off frequencies of the distributed LA and TA modes shown in Figure 1c, which are approximately ~20 GHz and ~10 GHz, respectively, are nearly the same to the Brillouin frequency shifts of the acoustic modes observed from the progesterone crystals at the backscattering geometry, which are plotted in Figure 2.

The Brillouin spectra can be curve-fitted by using the response function of the damped harmonic oscillator, which is approximated by the Lorentzian function in most cases. This response function should be convoluted with the instrumental Gaussian function of the interferometer, thus the resulting peak-fitting function is the Voigt function. Based on the peak-fitting procedure, the Brillouin frequency shift (*ν*_B_) and the full width at half maximum (FWHM, Γ_B_) can be derived as a function of temperature. The solid lines in Figure 1 denote the best-fitted results obtained from the curve-fitting procedure.

**Figure 3:**
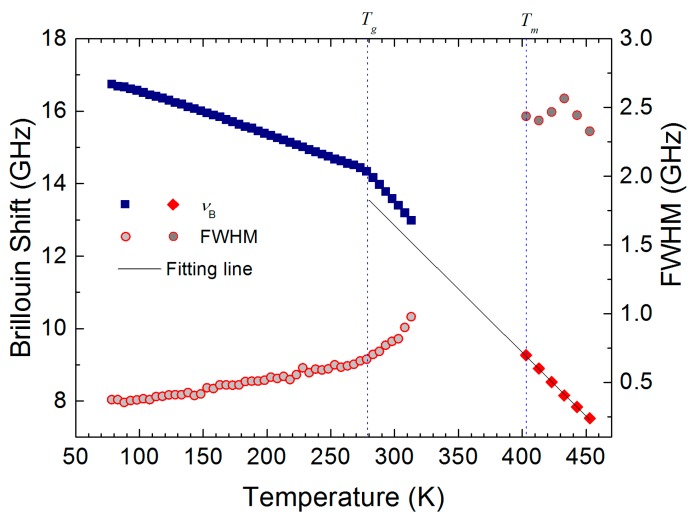
Temperature dependence of the Brillouin shift (*ν*_B_) and the full width at half maximum (FWHM, Γ_B_). The solid line denotes the result of the linear fitting for *ν*_B_ in the liquid phase. The two dotted lines show the glass transition temperature (*T_g_*) and the melting point (*T_m_*).

Figure 3 shows the temperature dependences of *ν*_B_ and Γ_B_ over the whole measurement temperature range from the glassy state to the liquid phase. The *ν*_B_ shows a monotonically decreasing behavior in the glassy state below 278 K at which its slope suddenly changes. This temperature corresponds to the glass transition temperature *T_g_* ~ 278 K detected by Brillouin scattering, which is nearly the same as the calorimetric *T_g_* (~279 K) [6]. The FWHM increases slowly in the glassy state upon heating, and begins to grow substantially above *T_g_*. The acoustic properties could not be obtained in a temperature range of 315–400 K because the progesterone was crystallized in this range. In the case of missing data between 320 K and *T_m_*, the sound velocity is expected to decrease nearly linearly and the FWHM to increase monotonically considering the general trends of amorphous pharmaceuticals. The *ν*_B_ and the Γ_B_ in the liquid phase are shown at temperatures above *T_m_*. The linear behavior of *ν*_B_ in the liquid phase was fitted by a linear function, resulting in *ν*_B_(*T*) = 23.34 − 0.035 × *T*. This is shown as a solid line in Figure 3. The FWHM in the liquid phase is much larger than the values in the glassy state. This behavior is similar to the cases of ibuprofen and ketoprofen, which exhibits the maximum of the FWHM in the liquid phase [20,26].

### 2.2. Analysis and Comparison

The *ν*_B_ and the Γ_B_ are related to the longitudinal sound velocity (*V*) and the acoustic absorption coefficient (*α*_B_), respectively, according to the following equations:

(1) $$V\;=\;\lambda \nu_{B}/2n,$$

(2) $$\alpha_{B}=\pi \Gamma_{B}/V.$$

**Figure 4:**
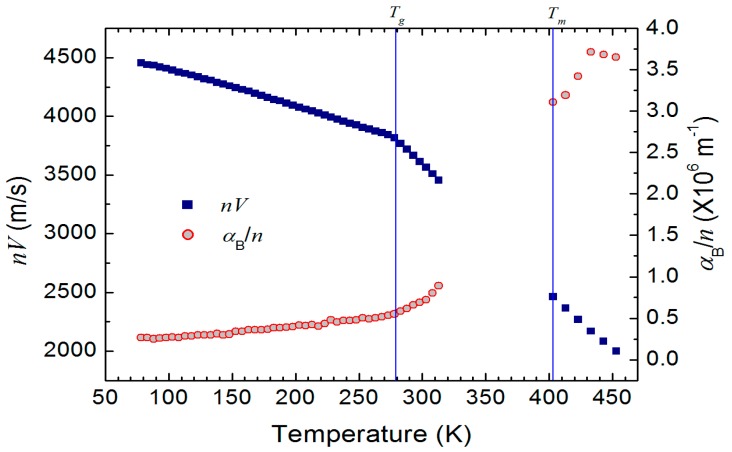
Temperature dependence of *nV* and *α*_B_/*n*. The glass transition temperature (*T_g_*) and the melting point (*T_m_*) are denoted by the vertical lines. The data are missing in a temperature range of 320 K–400 K due to crystallization.

In these equations, *λ* and *n* denote the laser wavelength, 532 nm in the present study, and the refractive index of progesterone, respectively. Because we have no information of the refractive index, the sound velocity and the absorption coefficient cannot be determined. In this case, one way to show the acoustic properties is to plot the *nV* and *α*_B_/*n* instead of *V* and *α*_B_. Figure 4 shows the temperature dependence of *nV* and *α*_B_/*n*. The average refractive index of progesterone single crystal is ~1.59 at room temperature [29]. If we assume this as the approximate refractive index of amorphous progesterone, the *V* and *α*_B_ at *T_g_* are ~2400 m/s and ~8.73 × 10^5^ m^−1^, respectively.

**Figure 5:**
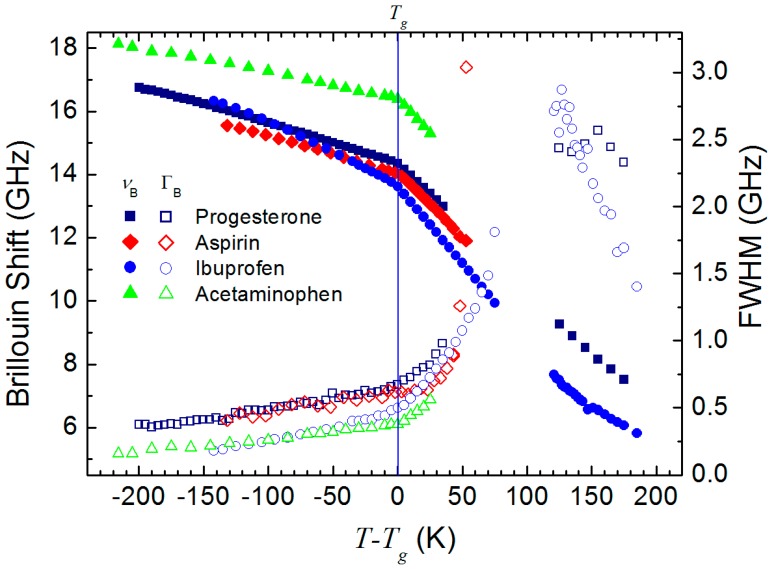
Comparison of the Brillouin shift (*ν*_B_) and the full width at half maximum (FWHM, Γ_B_) of four representative pharmaceuticals. The temperature scale is shown with respect to *T_g_*.

Figure 5 shows the comparison of the Brillouin shift and the FWHM of four pharmaceuticals, progesterone, aspirin [17], ibuprofen [20], and acetaminophen [23]. All these pharmaceuticals were investigated upon heating after melting and quenching to 77 K for vitrification. Figure 5 shows the common behaviors: for example, the Brillouin shift exhibits a sudden change in the slope at *T_g_*, above which the FWHM begins to grow significantly. These acoustic anomalies are caused by onset of a certain relaxation process in the supercooled liquid state. The relaxation process inducing the substantial change above *T_g_* can be analyzed by using a simple dispersion theory for the longitudinal velocity and absorption. If we assume a single relaxation time for the relevant relaxation process, the velocity *V* and the acoustic absorption coefficient *α*_B_ can be expressed by the following equations [30]:

(3) $$V^{2}=V_{0}^{2}+\left(V_{\infty }^{2}-V_{0}^{2}\right)\frac{\omega^{2}\tau^{2}}{1+\omega^{2}\tau^{2}}$$

(4) $$\alpha_{B}=\frac{1}{2V^{3}}\frac{\left(V_{\infty }^{2}-V_{0}^{2}\right)\omega^{2}\tau }{1+\omega^{2}\tau^{2}}$$

In these equations, *V*_0_ and *V_∞_* are fully-relaxed, low-frequency and unrelaxed, high-frequency sound velocity, respectively. *ω* is the angular frequency of the acoustic wave, and *τ* denotes the relaxation time of the relevant relaxation process responsible for the acoustic dispersion. By combining these two equations, we can derive the relaxation time *τ* as follows [30]:

(5) $$\tau =\frac{1}{2\pi \Gamma_{B}}\frac{\nu_{B}^{2}-\nu_{0}^{2}}{\nu_{B}^{2}}$$

where the linear relationship between *V* and *ν*_B_ shown in Equation (1) was used. The *ν*_0_ denotes the extrapolated value from the linear fitting for *ν*_B_ in the liquid phase, which is shown as a solid line in Figure 3. Because of the crystallization in the supercooled liquid phase, only a few data points for the relaxation time could be obtained at temperatures above *T_g_*.

**Figure 6:**
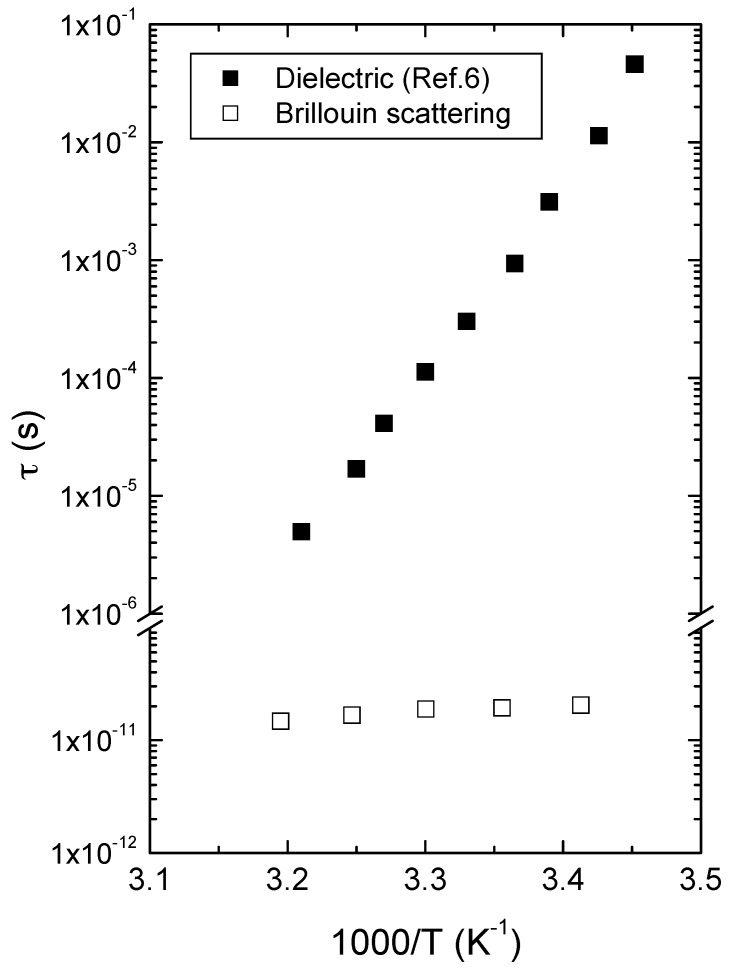
The relaxation times obtained from the dielectric and Brillouin scattering spectroscopies shown in the Arrhenius plot. The dielectric relaxation time was obtained from the impedance measurements in the frequency range of 10 Hz to 0.4 MHz, while the acoustic relaxation times from the longitudinal acoustic mode in the GHz range. The dielectric data were taken from [6].

It should be noted that non-Debye nature of the relaxation process could not be included in this analysis due to the lack of the relevant data and that the assumption of a single Debye relaxation process is for obtaining rough estimation of the order of the relaxation time of the relevant relaxation process near *T_g_*. Figure 6 shows the temperature dependence of the dielectric relaxation time of the *α*-process [6] and the fast relaxation process probed by Brillouin spectroscopy in the Arrhenius plot. The relaxation time of the fast process calculated by using Equation (5) exhibits the order of a few pico-seconds. This indicates that the acoustic dispersion at temperatures near *T_g_* is caused by some fast relaxation process. The difference between *ν*_B_ and *ν*_0_, shown in Figure 3, is related to this dispersion due to the fast process whose relaxation time is a few picosecond at temperatures above *T_g_*. Similar fast relaxation process was observed from the amorphous ketoprofen [26], which was attributed to some intramolecular motion of ketoprofen molecules. In contrast to the case of ketoprofen, the fast relaxation time shown in Figure 6 did not follow the Arrhenius relationship. This suggests that the acoustic dispersion observed from the sound velocity and the absorption cannot be solely attributed to the fast relaxation process and that some other relaxation process may also contribute to the acoustic dispersion. Inclusion of appropriate relaxation functions in the generalized longitudinal modulus is necessary, which requires the exact determination of the relaxation map, as well as the relaxation function of each process, of amorphous progesterone.

## 3. Discussion

Johari et al. reported detailed dielectric spectroscopic results on progesterone and other pharmaceuticals [6]. The dielectric constant (both real and imaginary parts denoted as *ε*’ and *ε*”, respectively) changes a little bit in the glassy state, while it grows rapidly as temperature increases above *T_g_*. The *ε*” exhibits two broad bumps in the glassy state of progesterone, which indicates secondary relaxation processes detectable in the kHz frequency range. On the other hand, the FWHM of the LA mode decreases almost linearly upon cooling below *T_g_*. This suggests that two dielectric relaxation processes do not couple to the density fluctuations of the longitudinal acoustic waves because of the large difference in the experimental frequency window of the two techniques. The growing Γ_B_ with increasing temperature toward *T_g_* from the glassy state is related to some local molecular mobility which becomes more active at higher temperature [16]. Remnant molecular motions in the rigid glassy structure are common in amorphous materials [31].

**Figure 7:**
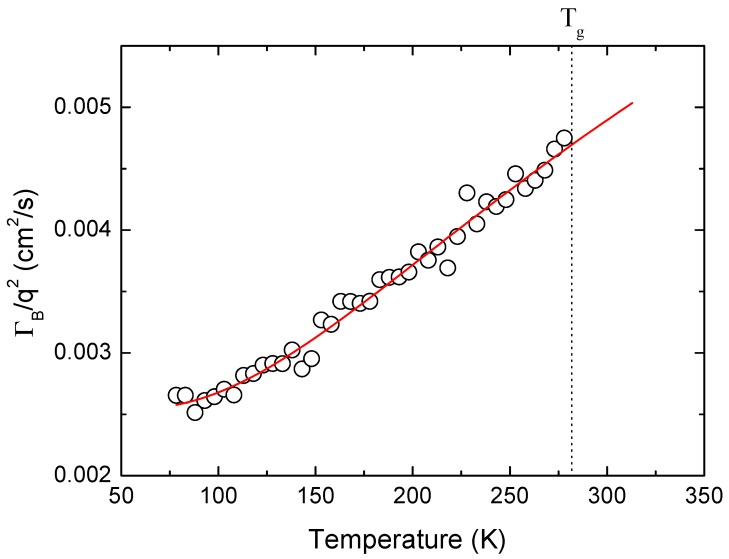
Temperature dependence of the longitudinal kinematic viscosity (open circles) along with the best-fitting result (red solid line) based on Equation (6). The vertical dotted line denotes the glass transition temperature.

These remnant motions are usually thermally-activated processes. These intramolecular motions affect the residual acoustic damping in the sub-*T_g_* region, and the longitudinal kinematic viscosity $\Gamma_{B}/q^{2}$ can be described by the following equation [16,32];

(6) $$\frac{\Gamma_{B}}{q^{2}}=\frac{\eta_{L}}{\rho }+\frac{\Delta }{\rho }\sin\left(\frac{\pi }{2}\beta \right)\omega_{LA}^{-\left(1+\beta \right)}\tau_{0}^{-\beta }\exp\left(-\frac{\beta E_{A}}{k_{B}T}\right)$$

where *ρ* is the density, *η_L_* is related to the instantaneous damping factor *γ*_0_ via *η_L_* = *ργ*_0_/*q*^2^. Δ is the relaxing part of the generalized elastic modulus, and *β* is the stretching parameter of the relaxation time distribution function of the intramolecular relaxation process. The angular frequency *ω_LA_* is 2*π* times *ν*_B_, and *k*_B_ is the Boltzmann constant. The attempted relaxation time *τ*_0_ and the activation energy *E_A_* are the relevant parameters that describe the sub-*T_g_* thermally-activated process. Most parameters in this equation are weakly temperature-dependent, and we assumed that these are constant parameters except for *βE_A_*. The refractive index of the amorphous progesterone is not available, thus we used the average refractive index of the progesterone crystal to obtain the room-temperature wavevector *q*. Based on this approach, the approximate value of *βE_A_* could be obtained. The best-fitted result is shown in Figure 7 where the temperature dependence of $\Gamma_{B}/q^{2}$ is shown in the sub-*T_g_* region along with the fitting line. The obtained value of *βE_A_*/*k*_B_ is 413 K, which indicates that *βE_A_* ~ 0.82 kcal/mol (3.4 kJ/mol). Available values of *βE_A_* can be found from several glass-forming materials that exhibit *βE_A_* of 140–610 K [33]. Unambiguous determination of *E_A_* is not possible due to the lack of the information of *β*. If the sub-*T_g_* thermally-activated process is purely single Debye type (*β* = 1) *E_A_* ~ 0.82 kcal/mol (3.4 kJ/mol). Considering the typical value *β* ~ 0.3 for amorphous polymers [16], the activation energy *E_A_* may be deduced to be 11 kJ/mol. This value is similar to the activation barrier for the hydroxyl or methyl group rotations [34]. This result clearly shows that the change in the damping behavior in the sub-*T_g_* region is attributed to side group rotations instead of single-molecule rotations or conformational change.

Once the temperature reaches *T_g_*, the amorphous progesterone enters into the so-called supercooled liquid or ultraviscous state. As viscosity decreases significantly in this state upon heating, the free volume in the amorphous progesterone is expected to increase, which allows more room for local molecular motions. More active molecular motions in enlarged free volume is the main origin of the substantial changes in the Brillouin shift and the FWHM at temperatures above *T_g_*.

Both components, *ε*’ and *ε*”, reach maxima at ~300 K and then decrease sharply upon further heating [6]. The dielectric maximum temperatures of both components are located in the supercooled or ultraviscous states where the Brillouin frequency shift decreases and the FWHM increases rapidly above *T_g_*. It is not surprising that the dielectric maximum temperature and the FWHM maximum temperature are different from each other because the former and the latter respond to quite different frequency ranges, that is, approximately 15 GHz for the former and 1 kHz for the latter. The precipitous drop of *ε*’ and *ε*” at temperatures above 315 K is related to the cold-crystallization of the ultraviscous progesterone, which is consistent with the appearance of the crystal peaks in the Brillouin spectrum (Figure 1c). Although the acoustic dispersion near *T_g_* is triggered by the fast relaxation process, we cannot exclude the possibility of the contribution of the secondary relaxation process to the acoustic dispersion at higher temperatures, because the previous dielectric study [6] showed that this process is active in the glassy state in the kHz frequency range. This indicates that the relaxation frequency of this process would possibly approach the Brillouin frequency window, resulting in coupling between the acoustic waves and the secondary relaxation process. However, this last suggestion should be proved by additional experimental measurements, such as broadband dielectric spectroscopy, on the secondary relaxation process in a wider frequency and temperature ranges.

The present study shows that the acoustic properties in the liquid phase of progesterone, that is, the sound velocity and the absorption coefficient do not represent fully-relaxed values. The high absorption coefficient of the LA waves in the liquid phase shows that significant damping still occurs in this phase. Intramolecular relaxation processes would certainly contribute to the acoustic dispersion in this liquid state because they persist at nearly all temperatures. However, this contribution is expected to be small considering the absorption coefficient in the glassy state solely caused by the intramolecular relaxation processes. Furthermore, Figure 6 shows that even the relaxation frequency of the structural *α*-relaxation process is expected to become comparable to the frequency of the LA waves as temperature increases. Based on the reported relaxation time of the structural relaxation process of the amorphous progesterone [6], the extrapolated relaxation time at 450 K becomes ~24 ps. This time scale is comparable to the Brillouin frequency window resulting in strong coupling between the structural relaxation process and longitudinal acoustic waves. Thus, we can conclude that *α*-relaxation process is the dominant contribution to the acoustic damping in the liquid phase, although contributions from fast and other secondary relaxation processes cannot be excluded. These contributions can be taken into account by considering generalized modulus where relaxing parts are included as phenomenological functions [16]. However, the crystallization of amorphous progesterone makes it difficult to consider correct functional forms for the modulus of each relaxation process.

Finally, we should consider the microscopic origin of the significant, persisting acoustic dispersion in the liquid progesterone, which is clearly represented by the maximum of the FWHM of the LA mode at ~420 K in the liquid state. Although many pharmaceuticals have hydrogen bonds in the structure, progesterone lacks hydrogen bonds in its crystal and liquid phases [35]. Shikii et al. reported that the large-scale aggregated chain structures, which were observed in many steroid compounds, were not observed in progesterone [35]. This suggests that a single-molecule nature is preserved in both supercooled liquid and liquid states of progesterone. Therefore, the molecular relaxation processes in both states are expected to be similar, and these active molecular motions as a whole may be responsible for the significant acoustic damping observed in the liquid phase. Pressure is another important variable that affects the molecular mobility significantly [36,37]. Combined investigation of amorphous pharmaceuticals by using both temperature and pressure changes will give us more insights into the microscopic nature of the peculiar molecular mobilities inherent in these disordered systems.

## 4. Materials and Methods

### 4.1. Materials

Progesterone crystalline powders (purity better than 99%) were purchased from Sigma-Aldrich Co. (Saint Louis, MO, USA) (CAS Number 57-83-0, product number P0130). It was dissolved in ethanol (purity > 99.9%, Merck, Kenilworth, NJ, USA) to carry out recrystallization. The solution was slightly supersaturated by considering the solubility of 1.13 × 10^−2^ g/mol at 40 °C and then cooled slowly to room temperature. Small crystals were obtained in terms of slow evaporation for 4–5 weeks. The morphology of the obtained crystals was the same to that reported in previous studies [11].

### 4.2. Methods

One or two progesterone crystals were inserted in a compact temperature controller (THMSE600, Linkam, Tadworth, UK) which was put under a microscope. Micro-Brillouin spectroscopy was applied to the sample for probing the acoustic properties. A modified microscope (BX-41, Olympus, Tokyo, Japan) was used for backscattering. The Brillouin spectrum was measured by using a conventional tandem six-pass Fabry-Perot interferometer (TFP-2, JRS Co., Zürich, Switzerland) and a diode-pumped solid state laser (Excelsior 532-300, Spectra Physics, Santa Clara, CA, USA). The laser was operated at 532 nm, and the power was 12 mW. The free spectral range of the interferometer was 30 GHz, and the Brillouin spectrum was acquired within ±27 GHz. A conventional photon-counting system combined with a multichannel analyser (1024 channels) was used to detect and average the signal. The details of the experimental setup can be found elsewhere [38,39].

The progesterone was heated from room temperature to 408 K above the melting point of ~403 K. Considering this high melting point, the present progesterone single crystal would be Form 1 which is thermodynamically more stable than Form 2. After the crystal was completely melted, the liquid progesterone was quenched to 77 K with a cooling rate higher than 10 K/s, which was enough to prevent crystallization and to form amorphous progesterone. Then, the Brillouin spectrum was measured with increasing temperature from 77 K. The typical acquisition time for measuring one spectrum was approximately one minute. The viscous melt became crystallized at ~315 K, and the crystallized progesterone was melted once more at 408 K. The Brillouin spectrum in the liquid phase was measured in a temperature range of 403–453 K, above which the sample seems to be degraded.

## 5. Conclusions

The acoustic properties of amorphous progesterone were investigated by Brillouin spectroscopy over a wide temperature range where glassy, ultraviscous, crystalline, and liquid states were observed sequentially upon heating. The sound velocity and the absorption coefficient of the longitudinal acoustic waves showed clear anomalies at the glass transition temperature. In addition, the transformation from the ultraviscous to the crystalline phase was clearly confirmed by the drastic change in the Brillouin spectra. A simple analysis based on a single-relaxation-time approximation revealed that a fast relaxation process in the pico-second range induces a significant acoustic dispersion in the ultraviscous state. The maximum of the acoustic damping located in the liquid phase indicated the existence of substantial contribution from the structural *α*-process. The present study showed that Brillouion spectroscopy is a powerful tool in the investigation of phase changes and fast relaxation dynamics of amorphous pharmaceuticals.

## References

[1] Hancock B.C., Zograf G. (1997). Characteristics and Significance of the Amorphous State in Pharmaceutical Systems. J. Pharm. Sci..

[2] Descamps M. (2016). Disordered Pharmaceutical Materials.

[3] Johari G.P., Pyke D. (2000). On the glassy and supercooled liquid states of a common medicament: Aspirin. Phys. Chem. Chem. Phys..

[4] Dyre J.C. (2006). The glass transition and elastic models of glass-forming liquids. Rev. Mod. Phys..

[5] Kothari K., Ragoonanan V., Suryanarayanan R. (2014). Influence of Molecular Mobility on the Physical Stability of Amorphous Pharmaceuticals in the Supercooled and Glassy States. Mol. Pharm..

[6] Johari G.P., Kim S., Shanker R.M. (2007). Dielectric Relaxation and Crystallization of Ultraviscous Melt and Glassy States of Aspirin, Ibuprofen, Progesterone, and Quinidine. J. Pharm. Sci..

[7] Johari G.P., Kim S., Shanker R.M. (2010). Dielectric Study of Equimolar Acetaminophen–Aspirin, Acetaminophen–Quinidine, and Benzoic Acid–Progesterone Molecular Alloys in the Glass and Ultraviscous States and Their Relevance to Solubility and Stability. J. Pharm. Sci..

[8] Campsteyn H., Dupont L., Dideberg O. (1972). Structure Cristalline et Moléculaire de la Progestérone, C_21_H_30_O_2_. Acta Crystallogr. Sect. B.

[9] Foresti S.E., Krajewski A., Mongiorgi R., Riva de Sanseverino L., Cameroni R. (1975). 4-Pregnen-3,20-Dione (Progesterone, Form 2). Cryst. Struct. Commun..

[10] Payne R.S., Roberts R.J., Rowe R.C., Docherty R. (1999). Examples of successful crystal structure prediction: Polymorphs of primidone and progesterone. Int. J. Pharm..

[11] Narayana Kalkura S., Devanarayanan S. (1988). Growth of progesterone crystals in silica gel and their characterization. J. Mater. Sci. Lett..

[12] Wang F., Wachter J.A., Antosz F.J., Berglund K.A. (2000). An investigation of solvent mediated polymorphic transformation of progesterone using in situ Raman spectroscopy. Org. Process Res. Dev..

[13] Cendejas-Santana G., Hinojosa-Torres J., Gastaño V.M. (2002). Progesterone crystallization from a solvent: A new procedure. Mater. Res. Innovat..

[14] Falcon J.A., Berglund K.A. (2004). In situ monitoring of antisolvent addition crystallization with principal components analysis of Raman spectra. Cryst. Growth Des..

[15] Lancaster R.W., Karamertzanis P.G., Hulme A.T., Tocher D.A., Lewis T.C., Price S.L. (2007). The polymorphism of progesterone: Stabilization of a ‘disappearing’ polymorph by co-crystallization. J. Pharm. Sci..

[16] Comez L., Masciovecchio C., Monaco G., Fioretto D. (2012). Progress in liquid and glass physics by Brillouin scattering spectroscopy. Solid Stat Phys..

[17] Ko J.-H., Lee K.-S., Ike Y., Kojima S. (2008). Elastic properties of aspirin in its crystalline and glassy phases studied by micro-Brillouin scattering. Chem. Phys. Lett..

[18] Kim T.H., Ko J.-H., Lee K.-S., Ike Y., Kojima S. (2009). Growth of Aspirin Single Crystals and the Temperature Dependence of Its Elastic Constants As Studied by Brillouin Scattering. New Phys. Sae Mulli.

[19] Ko J.-H., Kim T.H., Lee K.-S., Kojima S. (2011). Acoustic properties of aspirin in its various phases and transformation stages studied by Brillouin scattering. J. Non-Cryst. Solids.

[20] Ko J.-H., Kim T.H., Lee K.-S., Kojima S. (2011). Brillouin scattering study on crystalline and glassy states of anti-inflammatory racemic S(+)–R(−) ibuprofen. Chem. Phys. Lett..

[21] Kim T.H., Shibata T., Kojima S., Shin D.-M., Hwang Y.-H., Ko J.-H. (2014). Comparison of thermal and elastic properties of glassy racemic and enantiomorphic ibuprofen studied by Brillouin light scattering and modulated differential scanning calorimetry. Curr. Appl. Phys..

[22] Shin D.-M., Hwang Y.-H., Ko J.-H., Kojima S. (2015). Relaxation behaviors of enantiomorphic S-ibuprofen as revealed by dielectric and photon correlation spectroscopies. Curr. Appl. Phys..

[23] Kwon H.-J., Kim T.H., Ko J.-H., Hwang Y.-H. (2013). Relaxation phenomena in supercooled liquid and glassy acetaminophen studied by dielectric, photon correlation and Brillouin light scattering spectroscopies. Chem. Phys. Lett..

[24] Kim T.H., Ko J.-H., Kwon E.M., Jun J.-G. (2010). Micro-Brillouin Spectroscopy Applied to the Glass Transition of Anti-inflammatory Egonol. J. Opt. Soc. Korea.

[25] Ko J.-H., Kim T.H., Jung S.H., Jun J.-G. (2013). Study on the Possibility of Vitrification and Phase Transition in Decursinol Studied by Using Brillouin Light Scattering. New Phys. Sae Mulli.

[26] Shibata T., Takayama H., Kim T.H., Kojima S. (2014). Acoustic and thermal anomalies in a liquid–glass transition of racemic S(+)–R(−) ketoprofen. Chem. Phys. Lett..

[27] Vacher R., Boyer L. (1972). Brillouin scattering: A tool for the measurement of elastic and photoelastic constants. Phys. Rev. B.

[28] Ko J.-H., Kim D.H., Kojima S., Kim J.-H., Choo W.K. (2003). Brillouin Scattering Study on Polycrystalline Relaxor Ferroelectrics. Jpn. J. Appl. Phys..

[29] Watanabe A. (2002). A Trial Production of a Table of the Optical Crystallographic Characteristics of Crystalline Drugs Including Crystal Habits (Study of Crystalline Drugs by Means of a Polarizing Microscope. XIX. Yakugaku Zasshi.

[30] Torell L.M. (1982). Brillouin scattering study of hypersonic relaxation in Ca(NO_3_)_2_-KNO_3_ mixture. J. Chem. Phys..

[31] Kriegs H., Meier G., Gapinski J., Patkowski A. (2008). The effect of intramolecular relaxations on the damping of longitudinal and transverse phonons in polysiloxanes studied by Brillouin spectroscopy. J. Chem. Phys..

[32] Fioretto D., Scarponi F. (2009). Dynamics of a glassy polymer studied by Brillouin light scattering. Mater. Sci. Eng. A.

[33] Comez L., Pietrella M., Fioretto D., Monaco G., Scarponi F., Verbeni R. (2007). Brillouin-scattering study of the fast dynamics of m-toluidine. Philos. Mag..

[34] Schammé B., Mignot M., Couvrat N., Tognetti V., Joubert L., Dupray V., Delbreilh L., Dargent E., Coquerel G. (2016). Molecular Relaxations in Supercooled Liquid and Glassy States of Amorphous Quinidine: Dielectric Spectroscopy and Density Functional Theory Approaches. J. Phys. Chem. B.

[35] Shikii K., Sakamoto S., Seki H., Utsumi H., Yamaguchi K. (2004). Narcissistic aggregation of steroid compounds in diluted solution elucidated by CSI-MS, PFG NMR and X-ray analysis. Tetrahedron.

[36] Bryk T., Ruocco G., Scopigno T., Seitsonen A.P. (2015). Pressure-induced emergence of unusually high-frequency transverse excitations in a liquid alkali metal: Evidence of two types of collective excitations contributing to the transverse dynamics at high pressures. J. Chem. Phys..

[37] Grzybowska K., Capaccioli S., Paluch M. (2016). Recent developments in the experimental investigations of relaxations in pharmaceuticals by dielectric techniques at ambient and elevated pressure. Adv. Drug Deliv. Rev..

[38] Ko J.-H., Jeong M.-S., Lee B.W., Kim J.H., Ko Y.H., Kim K.J., Kim T.H., Kojima S., Ahart M. (2013). Pressure Dependence of Acoustic Properties of Liquid Ethanol by using High-pressure Brillouin Spectroscopy. Korean J. Opt. Photon..

[39] Oh S.H., Cho B.J., Jeong M.S., Ko J.-H. (2016). Evaluation of the isothermal curing process of UV-cured resin in terms of elasticity studied through micro-Brillouin light scattering. J. Inf. Disp..

